# Prevalence of scoliosis and congenital heart disease based on school screening in Jinghong City, Yunnan Province

**DOI:** 10.3389/fpubh.2025.1517542

**Published:** 2025-02-25

**Authors:** Genghao Qian, Li Zhang, Zhi Zhao, Yingsong Wang, Jiang Lu, Ni Bi, Xiaochen Yang, Weijie Xie, Zhiyue Shi, Tao Li, Zhaoquan Zhang, Zhibo Song

**Affiliations:** ^1^Orthopedics, Second Affiliated Hospital of Kunming Medical University, Kunming, China; ^2^Medical Department, Yunnan Fuwai Cardiovascular Disease Hospital, Kunming, China

**Keywords:** scoliosis, congenital heart disease, epidemiology, school screening, children

## Abstract

**Objective:**

To investigate the prevalence of scoliosis and congenital heart disease (CHD) in the same area and to explore the relationship between them according to a joint school screening.

**Methods:**

All students aged 6–15 years in 20 schools in Jinghong City, Yunnan Province, China was screened for scoliosis and CHD. Scoliosis screening completed through the Adam's forward bending test with scoliometer measurement, and CHD screening completed through auscultation combined with portable echocardiography (ECHO). The gender, age, distribution of ethnic groups, types of CHD, angle of trunk rotation (ATR) and location of scoliosis were recorded. The severity was divided into 3 grades by ATR. Then the relationship between scoliosis and CHD was analyzed.

**Results:**

A total of 17,134 students was screened with a prevalence of suspected scoliosis of 1.7% (298 students), and the prevalence of suspected scoliosis in female was higher than that in male (2.4 vs. 1.2%, *P* < 0.001), which increased with age (*P* < 0.01). The prevalence of suspected scoliosis was no different among ethnic groups (*P* > 0.05). The severity of scoliosis was mainly grade 1 (68.5%), followed by grade 2 (27.2%) and grade 3 (4.4%). And scoliosis was mainly located in lumbar (37.6%). The prevalence of CHD was 3.15‰, and there was no difference in the prevalence of CHD between different gender, age and ethnic groups (*P* > 0.05). The most common type of CHD was atrial septal defect (27.78%), followed by ventricular septal defect (16.67%). There was only one CHD student in 298 suspected scoliosis students.

**Conclusion:**

The prevalence of suspected scoliosis among primary school students was 1.74%, while the prevalence of congenital heart disease was 3.15‰ in Jinghong City, Yunnan Province. And the incidence of CHD in patients with mild suspected scoliosis was low and close to that in normal population.

## Introduction

Scoliosis is indeed a complex three-dimensional deformity of the spine, characterized by vertebral rotation and imbalances in the coronal, sagittal, and axial planes. It can be broadly categorized into idiopathic scoliosis (IS) and non-idiopathic scoliosis (Non-IS) (1).

Both the musculoskeletal and circulatory systems originate from the mesoderm, with numerous genes and signaling pathways collaboratively regulating cell differentiation and organ development in these systems (2–6). Environmental factors, such as hypoxia during pregnancy, vitamin deficiencies, alcohol consumption, and maternal diabetes, have also been implicated in the co-occurrence of scoliosis and congenital heart diseases (CHD) (7–10). These two developmental processes are interconnected: the hypoxia environment in patients with cyanotic congenital heart disease may serve as a risk factor for the onset and progression of scoliosis (11). The interplay of these extrinsic and intrinsic factors likely contributes to the higher prevalence of congenital heart disease among scoliosis patients compared to the general population. Previous clinical studies have reported that the incidence of CHD in patients with idiopathic scoliosis (IS) ranges from 4.14 to 8.75% (12–14), while the incidence in patients with congenital scoliosis (CS) ranges from 6.95 to 26% (15). Investigating the relationship between these diseases can help uncover shared genetic variations and enhance our understanding of their common etiology. Furthermore, such research can improve the comprehensive management of patients, ensuring they receive holistic evaluation and treatment. By understanding the prevalence and influencing factors of these comorbidities, healthcare systems can better allocate resources and reduce long-term healthcare costs.

Previous studies have primarily focused on patients with severe scoliosis requiring surgical intervention, often conducted in hospital settings. However, there is a lack of population-based studies examining the incidence of congenital heart disease associated with scoliosis in children and adolescents. To address this gap, this study investigated and analyzed the prevalence of scoliosis and CHD among 17,134 primary school students in Jinghong City, Yunnan Province. The findings aimed to provide valuable insights for research and prevention strategies targeting scoliosis and CHD in children and adolescents.

## Materials and methods

The screening was conducted in Jinghong City, located in the Xishuangbanna Dai Autonomous Prefecture of Yunnan Province. Using a random number table method, 17,134 students from 20 primary schools were randomly selected from a total of 52 primary schools in the city. The inclusion criterion was all students aged 6–15 years in 20 primary schools included in this study, while the exclusion criteria were: (1) Unable to cooperate in completing Adam's forward bending test, cardiac auscultation or echocardiography examination, (2) Unwilling to participate in scoliosis screening or congenital heart disease screening.

Prior to the screening, detailed information about the screening process was communicated to teachers and students in the target schools. Informed consent was obtained from teachers, students and their parents. The study received approval from the Ethics Review Committee of the Second Affiliated Hospital of Kunming Medical University (PJ-2021-100) and the Ethics Review Committee of Fuwai Cardiovascular Hospital of Yunnan Province (IRB2017-BG-028).

**Figure 1:**
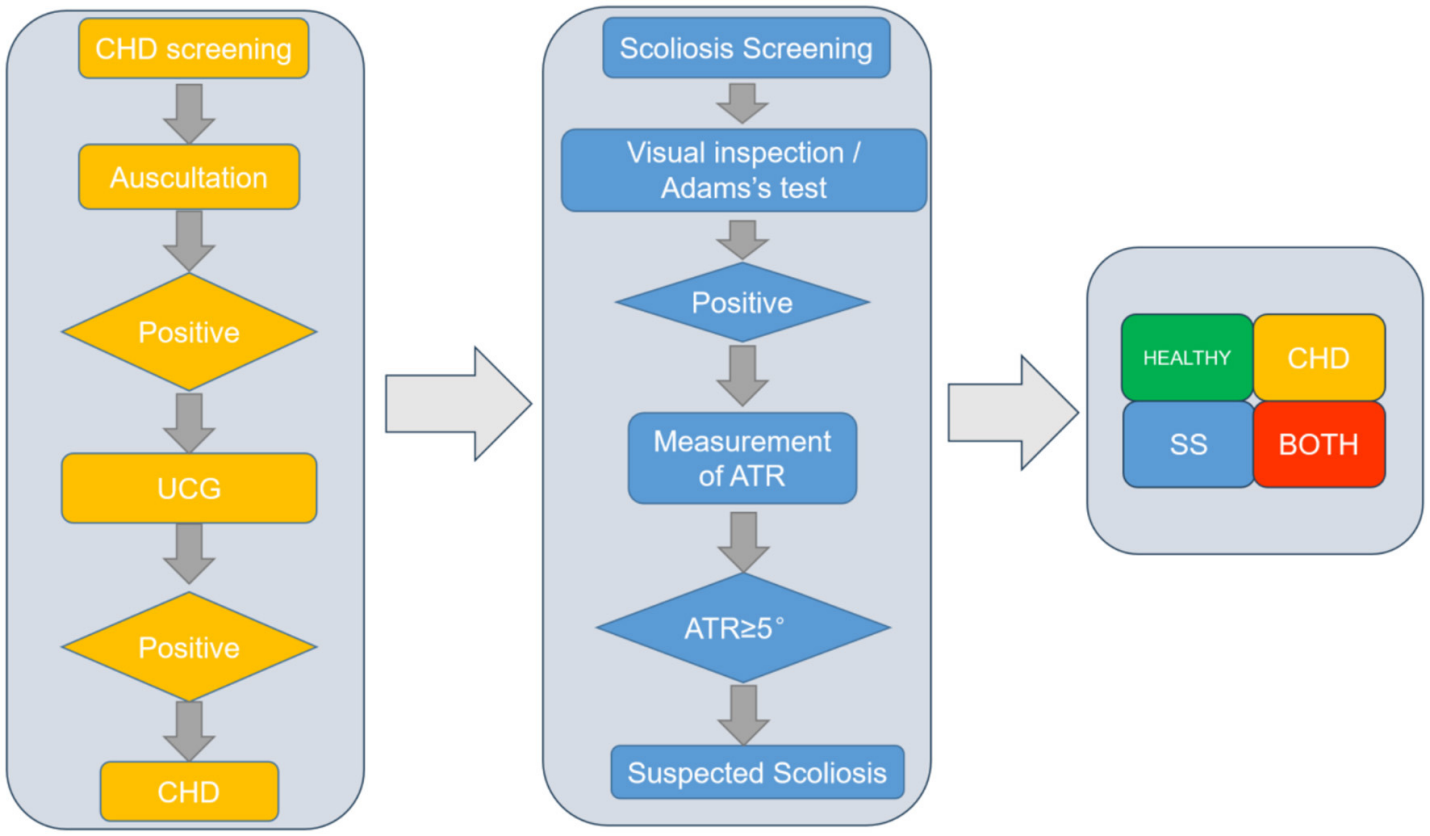
Flow chart of scoliosis and CHD joint screening.

The scoliosis screening team was composed of spine surgeons from our department, graduate students, as well as orthopedic surgeons and nurses from local hospitals. All personnel underwent standardized training based on the Chinese guidelines named “Screening of Abnormal Spinal Curvature in Children and Adolescents” (16). The Scoliosis Research Society (SRS), the American Academy of Orthopedic Surgeons (AAOS), and several relevant U.S. academic institutions have collectively endorsed that well-trained screeners conducting an effective scoliosis screening program can facilitate early detection of adolescent scoliosis. During the screening process, school-aged adolescents were assessed using the Adams forward bending test and the trunk rotation angle (ATR) measured with a scoliometer (17). A comprehensive evaluation was performed utilizing multiple methods, including visual inspection, Adam's forward bending test, and ATR measurement. Visual inspection focused on observing the child's posture, shoulder height, scapular symmetry, lumbar contour, iliac crest height, and the alignment of the spinous process line relative to the posterior midline. For Adams's test, subjects were instructed to bend forward at a 90° angle, with any asymmetry in the back indicating a positive result. ATR measurements were conducted for subjects who tested positive on either the Adams's test or visual inspection (Figure 1). An ATR of ≥5° was considered indicative of scoliosis, with severity graded as follows: Grade I (5° ≤ ATR < 7°), Grade II (7° ≤ ATR < 10°), or Grade III (ATR ≥ 10°) (16).

Congenital heart disease screening personnel consisted of doctors and sonographers from Fuwai Cardiovascular Hospital of Yunnan Province. All personnel were screened and trained according to the Handbook of Congenital heart Disease Screening for Children in Community (18). The diagnostic criteria and disease classification of congenital heart disease were referred to the “Diagnostic Criteria for Congenital Heart Disease” (19). The screening of congenital heart disease included initial screening and diagnosis. Cardiac auscultation was performed for all students in the primary screening. If there was no abnormality in the initial auscultation, it was negative. Subjects with a previous diagnosis of congenital heart disease were considered as the confirmed population and reported to the staff directly (Figure 1).

Demographic information such as gender, age, ethnicity, height, weight, school, and grade was collected. For scoliosis, ATR value, site of abnormality, and direction of the spinal curve were recorded. Diagnostic information for students with congenital heart disease was also collected.

Data analysis was performed using SPSS 27.0 software, with enumeration data described by the number of cases and detection rate. The detection rate was expressed as a percentage (%). The screening population was categorized based on gender, age, and ethnicity, and the detection rate between groups was compared using the χ^2^ test. A significance level of *P* < 0.05 was considered statistically significant in all tests.

## Result

### Detection of scoliosis

**Figure 2:**
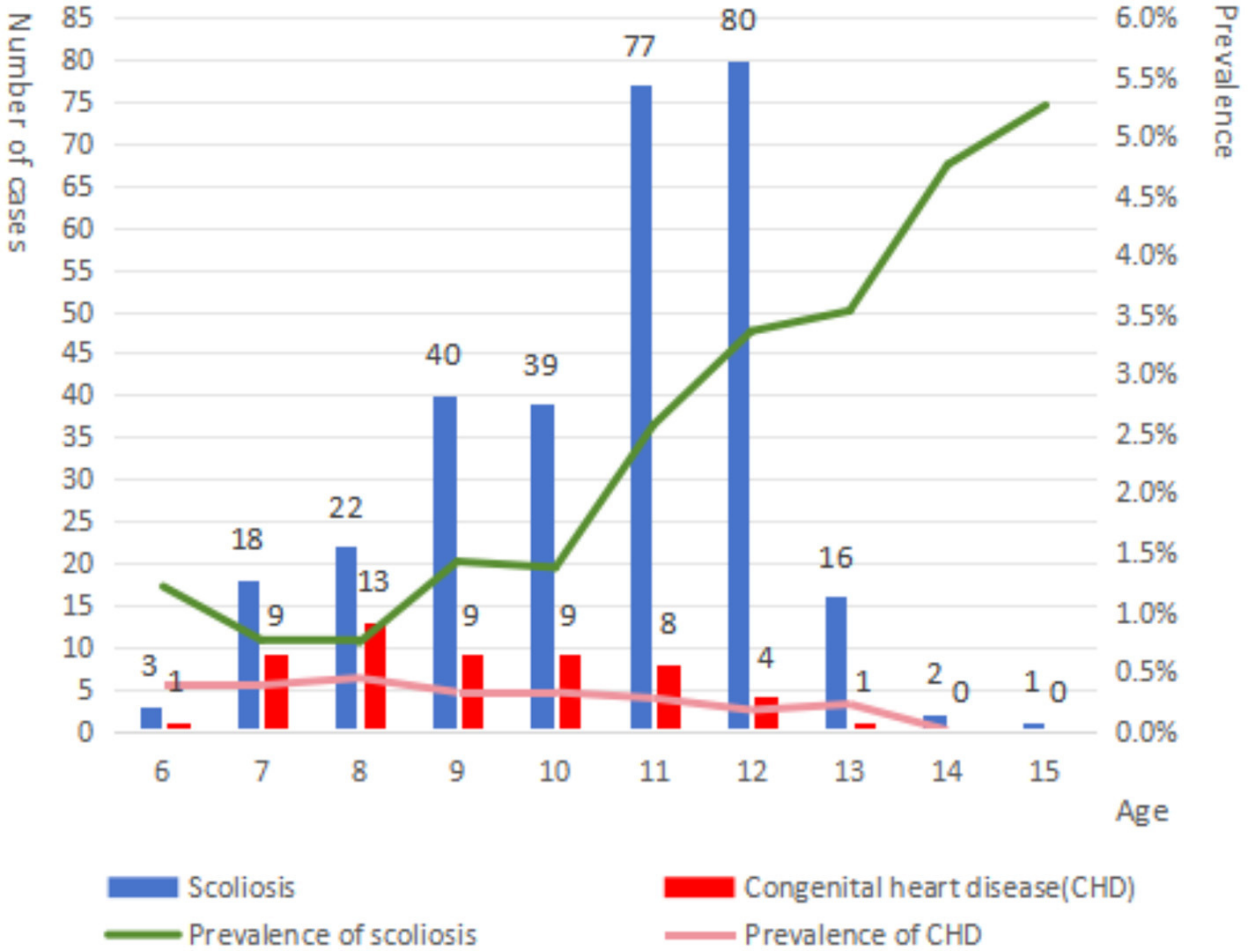
Changes in positive rates of scoliosis and congenital heart disease with age.

There were 8,835 males and 8,299 females included in this study. The prevalence of suspected scoliosis was 1.74%. The prevalence in male was 1.15%, which was significantly lower than that in female 2.36% (*P* < 0.001). In terms of age distribution, the prevalence of suspected scoliosis increased with age, especially after the age of 10 years (Figure 2). And the prevalence of suspected scoliosis rate in adolescents (11–15 years old) was significantly higher than that in children (6–10 years old) (*P* < 0.001). There was no significant difference in the prevalence of suspected scoliosis among children and adolescents of different ethnic groups (*P* = 0.057, Table 1).

**Table 1:** Detection of scoliosis.

**Project**		**Total number of screenings**	**SS**	**Prevalence of SS (%)**	**χ^2^**	***P***
Gender	Male	8,835	102	1.15	36.49	< 0.001
	Female	8,299	196	2.36		
Age	Children (6–10 Y)	11,239	122	1.09	81.69	< 0.001
	Adolescents (11–15 Y)	5,895	176	2.99		
Ethnic	Han	5,106	104	2.04	7.52	0.057
	Dai	4,171	70	1.68		
	Hani	3,672	47	1.28		
	Others	4,185	77	1.84		

### The ATR grade of scoliosis and the distribution of scoliosis sites

A total of 298 cases were suspected with scoliosis, including 204 cases (68.45%) of scoliosis grade I, 81 cases (27.18%) of scoliosis grade II, and 13 cases (4.36%) of scoliosis grade III. The thoracic segment, thoracolumbar segment and lumbar segment accounted for 35.57%, 26.84%, and 37.58%, respectively. There was no statistically significant difference in the distribution of the severity of scoliosis between different sites (*P* = 0.482, Table 2).

**Table 2:** ATR grading of scoliosis and distribution of suspected scoliosis sites.

**ATR grade**	**Thoracic segment**	**Thoracolumbar segment**	**Lumbar segment**	**Total number**	**χ^2^**	***P***
Grade I	77 (25.83%)	52 (17.45%)	75 (25.17%)	204 (68.45%)	3.476	0.482
Grade II	26 (8.72%)	22 (7.38%)	33 (11.07%)	81 (27.18%)		
Grade III	3 (1.01%)	6 (2.01%)	4 (1.34%)	13 (4.36%)		

### Detection of congenital heart disease

A total of 54 patients with congenital heart disease were detected, including 27 males and 27 females, with an overall prevalence of 0.315%, and the prevalence was not significantly different in gender ratio, age distribution, and ethnic distribution (Table 3). The prevalence of atrial septal defect (27.78%), ventricular septal defect (16.67%), pulmonary valve stenosis (12.96%) and aortic valve malformation with stenosis (12.96%) were the top five congenital heart diseases. There were 7 cases of postoperative children, and the specific diagnosis was unknown (Table 4).

**Table 3:** Detection of congenital heart disease.

**Project**		**Total number of screenings**	**CHD**	**Prevalence of CHD (%)**	**χ^2^**	***P***
Gender	Male	8,835	27	0.31	0.05	0.818
	Female	8,299	27	0.33		
Age	Children (6–10 Y)	11,239	41	0.36	2.56	0.109
	Adolescents (11–15 Y)	5,895	13	0.22		
Ethnic	Han	5,106	17	0.33	2.4	0.494
	Dai	4,171	17	0.41		
	Hani	3,672	8	0.22		
	Others	4,185	12	0.29		

**Table 4:** Distribution of congenital heart disease types.

**Project**	**Cases (percentage)**
Atrial septal defect (ASD)	15 (27.78%)
Ventricular septal defect (VSD)	9 (16.67%)
ASD + ASD	1 (1.85%)
Patent foramen ovale (PFO)	5 (9.26%)
Congenital pulmonary valve stenosis	7 (12.96%)
Bicuspid aortic valve	7 (12.96%)
Double-orifice mitral valve	2 (3.70%)
Mirrored dextrocardia	1 (1.85%)
Postoperative CHD (unspecified)	7 (12.96%)

### Scoliosis and congenital heart disease

**Figure 3:**
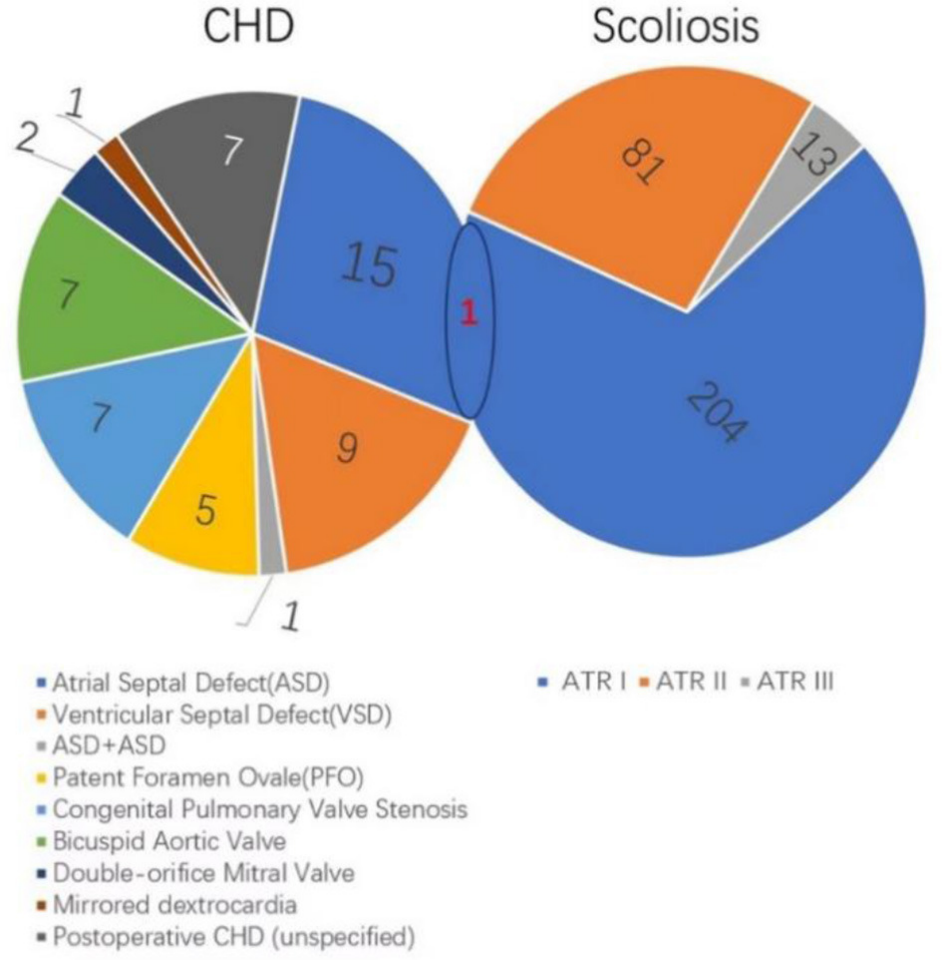
Co-occurrence of scoliosis and congenital heart disease.

In this study, 298 cases were screened as suspected scoliosis, 54 cases were diagnosed with congenital heart disease, and 1 case with scoliosis combined with congenital heart disease (Table 5; Figure 3).

**Table 5:** Relationship between scoliosis and congenital heart disease.

	**No scoliosis**	**SS**	**χ^2^**	***P***
No CHD	16,783	297	0.004	0.949
CHD	53	1		

## Discussion

The prevalence of suspected scoliosis among primary school students in Jinghong area.

This study represents the first large-scale epidemiological investigation of suspected scoliosis prevalence and its influencing factors in Jinghong City, Southwest China. The prevalence of suspected scoliosis among students in Jinghong City was 1.74%, which was comparable to the 1.50% reported in a 2016 Brazilian study of children and adolescents aged 6–18 (20). However, this prevalence was higher than those reported in the United States (0.2%) (21) and Japan (0.87%) (22), yet lower than the rates observed in our team's 2021 screening of primary and secondary school students aged 6–18 in Dali Bai Autonomous Prefecture (23), as well as in other regions of China, such as Chongming, Wuxi, and Zhejiang (24–26).The relatively lower prevalence in Jinghong may be attributed to the study's focus on primary school students aged 6–10, who constitute ~65% of the screened population. Scoliosis occurring before the age of 10, termed early-onset scoliosis (EOS), has a reported annual incidence of 0.019% in Asian children under 10 years old (27). Additionally, the most common form of scoliosis, adolescent idiopathic scoliosis (AIS), typically progresses during adolescence, with incidence rates increasing significantly during this period. Adolescence generally occurs between ages 9–14 in girls and 10–15 in boys (28–30), which aligns with our findings of a notable increase in positive rates after age 10. Furthermore, altitude may play a role in scoliosis prevalence. While previous studies suggest that altitudes above 4,500 m are a risk factor for scoliosis (31), Jinghong City's average altitude of 552 m is significantly lower, potentially contributing to the observed lower prevalence.

Current research has established significant gender and age differences in scoliosis incidence. Although the initial screening positive rate does not equate to the true diagnostic rate of scoliosis, it remains a valuable epidemiological indicator. In this study, the incidence was 1.15% for males and 2.36% for females, with males exhibiting a significantly lower incidence than females, consistent with previous findings (20–26). Despite the study's focus on primary school students aged 6–15, the results align with the established trend of increasing scoliosis incidence with age within a specific range (23–26). The observed higher positive rate in certain age groups may be attributed to the relatively small sample size in this study.

The survey was conducted in Jinghong City, a region with a high proportion of ethnic minorities, predominantly the Dai and Hani ethnic groups. The data revealed a difference in the scoliosis positivity rate between the Han ethnic group and ethnic minorities, although this difference was not statistically significant (*P* = 0.057). This finding may be attributed to the relatively small sample size of certain ethnic groups participating in the screening or potential variations in genetic factors, socioeconomic status, lifestyle habits, and health awareness among different ethnic groups in the study area (32).

### ATR grade of scoliosis and distribution of scoliosis sites

The degree of back asymmetry caused by scoliosis, while not perfectly correlated with the severity of spinal curvature or vertebral rotation, shows a significant association with the Cobb angle and the angle of trunk rotation (ATR) measured by scoliometer. A higher ATR angle was indicative of a greater likelihood and severity of scoliosis (33). In this study, 204 students (68.45%) with an initial positive screening result had Grade I ATR scores, while only 13 students (4.36%) had Grade III ATR scores, consistent with the distribution observed in other screening studies (34, 35). This suggests that the majority of initially positive cases in this study involved mild scoliosis, which is often clinically subtle. A key objective of scoliosis screening is to identify such mild cases early, enabling timely interventions to mitigate disease progression.

Among the 298 initially positive cases, 4 exhibited ATR >5° in both thoracic and lumbar segments. The distribution of higher ATR degrees was as follows: thoracic scoliosis in 106 cases (35.57%), thoracolumbar scoliosis in 80 cases (26.84%), and lumbar scoliosis in 112 cases (37.58%). While studies on the predominant location of scoliosis vary—some identifying the thoracic segment as the most common site and others the lumbar segment—there is a consensus that single-curve scoliosis is the most frequently detected pattern in screening programs (36–39).

### Cardiac abnormalities in students with suspected scoliosis

This study utilized portable echocardiography to diagnose congenital heart disease (CHD), identifying 54 cases with a prevalence rate of 0.315%. Notably, only one student (0.34%) suspected with scoliosis was found to have concomitant CHD, a proportion significantly lower than the 3.3–28.57% reported in previous studies of scoliosis patients requiring surgical intervention (12–15). This discrepancy may be attributed to several factors:

1. Population Characteristics:
Among the 11,239 students aged 6–10 screened, the prevalence of early-onset scoliosis (EOS) was estimated at 0.077%, corresponding to ~10 cases. Adolescent idiopathic scoliosis (AIS) accounts for ~80% of scoliosis cases identified in school screenings, with EOS representing a minor subset. EOS is characterized by rapid Cobb angle progression, larger final curvature, and a higher likelihood of coexisting congenital anomalies. For instance, Mason et al. reported that nearly 75% of EOS patients had at least one congenital anomaly, and 50% had two or more (40). Specific syndromes, such as neurofibromatosis type 1 (17q11.2 deletion) and Sotos syndrome, are often associated with CHD and other systemic abnormalities (41, 42). The low prevalence of CHD in this study's scoliosis population may reflect the predominance of AIS over EOS in school-based screenings.
2. Disease Severity and Selection Bias:
The suspected scoliosis cases identified in this study were primarily in the early stages, with fewer severe cases requiring surgical intervention. In contrast, previous studies focused on severe scoliosis patients, who are more likely to exhibit comorbidities such as CHD. Children with CHD may be predisposed to severe scoliosis due to chronic hypoxia, which promotes reactive oxygen species accumulation and mitochondrial dysfunction, potentially exacerbating spinal deformity (8, 43). Similarly, EOS patients are more likely to progress to severe scoliosis requiring surgery (44). The low proportion of older students in this study may have introduced bias, as severe cases often present later in adolescence.
3. Diagnostic Limitations:
While the combination of visual inspection, Adams test, and trunk rotation angle (ATR) measurement offers high sensitivity and specificity, it was not the gold standard for scoliosis diagnosis. This may have led to the inclusion of healthy individuals among suspected cases, potentially underestimating the true prevalence of scoliosis with CHD.
4. Regional and Methodological Factors:
The incidence of CHD in the study area (3.19‰) was lower than that in regions such as Guangdong and Shandong (45–47), which may contribute to the observed low prevalence of scoliosis with CHD.

In addition to the limited diagnosis of scoliosis mentioned above, this study also has the following limitations: This study mainly focuses on primary school students, with a relatively low proportion of adolescents. However, scoliosis is more common in adolescents aged 13–14, which may lead to a lower prevalence of scoliosis combined with congenital heart disease. And it is not yet known whether the children surveyed in this study will develop scoliosis with age, which may also lead to a lower proportion of scoliosis combined with CHD. Although this study surveyed 17,134 primary school students, the incidence was low and the number of patients was small, making it difficult to conduct in-depth analysis of risk factors for scoliosis combined with CHD. After comparing and analyzing previous studies with the results of this study, we speculate that patients with scoliosis combined with CHD are more likely to progress. However, long-term follow-up is needed for verification. We will expand the sample size of the survey and conduct follow-up on key populations to further clarify the relationship between scoliosis and CHD.

## Conclusion

In summary, the prevalence of suspected scoliosis among primary school students was 1.74%, while the prevalence of congenital heart disease was 3.15‰ in Jinghong City, Yunnan Province. And the incidence of CHD in patients with mild suspected scoliosis was low and close to that in normal population. Patients with scoliosis combined with CHD may be more prone to sustained progression.

## Data Availability

The raw data supporting the conclusions of this article will be made available by the authors, without undue reservation.
